# Mental practice in postgraduate training: a randomized controlled trial in mastoidectomy skills

**DOI:** 10.1186/s40463-016-0162-2

**Published:** 2016-09-15

**Authors:** Anne Conlin, Jane Lea, Manohar Bance, Neil Chadha, Shaun Kilty, Frederick Kozak, Julian Savage, Ravindar Sidhu, Joseph Chen, Brian D. Westerberg

**Affiliations:** 1 Division of Otolaryngology-Head and Neck Surgery, B. C. Rotary Hearing and Balance Centre at St Paul’s Hospital, University of British Columbia, Providence 2 - 1081 Burrard Street, Vancouver, BC V6Z 1Y6 Canada; 2 Division of Otolaryngology-Head & Neck Surgery, Dalhousie University, Halifax, NS Canada; 3 Division of Pediatric Otolaryngology-Head and Neck Surgery, B.C. Children’s Hospital, Vancouver, BC Canada; 4 Department of Otolaryngology-Head & Neck Surgery, The Ottawa Hospital, Ottawa, ON Canada; 5 Ottawa Hospital Research Institute, Ottawa, ON Canada; 6 Division of Vascular Surgery, University of British Columbia, Vancouver, BC Canada; 7 Department of Otolaryngology-Head & Neck Surgery, University of Toronto, Toronto, ON Canada; 8 Sunnybrook Health Sciences Centre, Toronto, ON Canada

**Keywords:** Mastoid, Imagery, Motor skills, Education, Medical, Graduate

## Abstract

**Background:**

Mental practice, the cognitive rehearsal of a task in the absence of overt physical movement, has been successfully used in teaching complex psychomotor tasks including sports and music, and recently, surgical skills.

The objectives of this study were, 1) To develop and evaluate a mental practice protocol for mastoidectomy 2) To assess the immediate impact of mental practice on a mastoidectomy surgical task among senior Otolaryngology─Head & Neck Surgery (OHNS) residents.

**Method:**

Three expert surgeons were interviewed using verbal protocol analysis to develop a mastoidectomy mental practice script. Twelve senior Residents from Canadian training programs were randomized into two groups. All Residents were video-recorded performing a baseline mastoidectomy in a temporal bone lab. The intervention group received mental practice training, while the control group undertook self-directed textbook study. All subjects were then video-recorded performing a second mastoidectomy. Changes in pre- and post-test scores using validated expert ratings, the Task Specific Evaluation of Mastoidectomy and the Global Evaluation of Mastoidectomy, were statistically analyzed.

**Results:**

A mental practice script was successfully developed based on interviews of three expert surgeon-educators. Task Specific Evaluation and Global Evaluation scores increased in both the mental practice and textbook study groups; there was no significant difference between the two groups in the change in scores post-intervention. There was a high and statistically signficant correlation between evaluators on the outcome measures.

**Conclusions:**

We were not able to demonstrate a significant difference for the benefits of mental practice in mastoidectomy, possibly due to the sample size. However, mental practice is a surgical education tool which is portable, accessible, inexpensive and safe.

**Electronic supplementary material:**

The online version of this article (doi:10.1186/s40463-016-0162-2) contains supplementary material, which is available to authorized users.

## Background

Multiple factors may have a negative impact on technical training of surgical Residents, including time constraints in operating rooms, financial limitations, patient safety concerns and the ratio of patients to Residents. Shifting patterns of health care delivery and working hours restrictions have led to a significant reduction in training opportunities [1]. Concurrently, there has been an increased awareness of medical errors and a recognition of the deficiencies in evaluating the performance and competence of practicing surgeons [2]. Poor technical performance and preventable surgical complications may contribute to a large proportion of medical errors [3].

The ideal method of teaching surgical Residents must be effective, safe, accessible and affordable. Current trends in surgical education have demonstrated superior outcomes among Residents who engage in learning activities such as computer-based video training and the use of simulators [4–11]. While these have expedited the rate of acquisition of surgical skills, they are procedure-specific, expensive and often difficult to access.

Mental practice is “the cognitive rehearsal of a task in the absence of overt physical movement” [12]. In essence, the performer systematically uses mental imagery to rehearse a skill. Mental practice has been used successfully in teaching and rehearsing complex psychomotor tasks in several domains, including sports and music [12–14] and recently, surgical skills acquisition [15, 16]. The process of mental practice typically involves a period of relaxation exercises, followed by an expert educator reciting a mental imagery script with emphasis on visual, kinesthetic and cognitive cues [15, 16]. Meta-analyses have indicated that mental practice is effective for all types of tasks, but is most effective for tasks that have considerable cognitive components [12, 17].

The purpose of this pilot study was to assess the impact of mental practice on Residents’ surgical skills during mastoidectomy, a challenging surgical procedure both to learn and to teach. The specific objectives of the study were: first, to develop a mental practice protocol for mastoidectomy created from expert educators’ instructional input and further tailored based on actual residents’ feedback; and second, to quantitatively and qualitatively assess the impact of mental practice on mastoidectomy surgical skills among senior residents, specifically in Post-Graduate Year 5 (PGY-5), from training programs across Canada.

## Methods

### Development of the mental practice protocol

To develop the mental practice script, semi-structured focused interviews were held with expert practicing surgeons (MB, JC, BDW). Using a cognitive walk-through technique and verbal protocol analysis [18] the expert surgeons identified the steps in performing a mastoidectomy. Specifically, the surgeons were prompted to report the visual, cognitive and kinesthetic cues involved in the procedure. Interviews were audiotaped and transcribed. The transcripts were then coded by one coder (AC) using emergent theme analysis on a coding framework [19], based on a previously published, validated framework of the steps for completion of a mastoidectomy, geared towards Residents [20]. The findings from the three interviews were merged to create a single mental practice script.

To evaluate the mental practice protocol, all six Residents in Post-Graduate Years 3–5 in the Otolaryngology-Head & Neck Surgery (OHNS) program at the University of British Columbia were enrolled. All subjects had participated in mastoidectomy surgery in both the temporal bone laboratory and operating rooms. Informed consent was obtained from all participants. After a brief introduction to the field of mental practice, the subjects listened to an audio copy of the mental practice script. The subjects assessed the quality of the mental practice script using the standardized Mental Imagery Questionnaire-Revised (MIQ-R).

### Impact of mental practice on mastoidectomy skills

The impact of the mental practice protocol on mastoidectomy skills was assessed among 12 OHNS Residents attending an annual national specialty review course in Halifax, Nova Scotia. To be included in the study, subjects had to be enrolled in PGY-5 (the final training year for OHNS in Canada) of a Canadian OHNS Residency program and provide informed consent. Residents from UBC involved in the evaluation phase of the study were excluded. Individuals who had previously trained or practiced in a foreign country, and were currently enrolled in a Canadian OHNS Residency program, were also excluded from the study. A total of 36 individuals were enrolled in the review course, and all participants were contacted as potential subjects. Thirteen individuals were excluded due to not meeting inclusion criteria. A further eleven potential subjects declined to participate. The remaining 12 individuals were enrolled in the study in parallel treatment arms with equal allocation.

All subjects completed a baseline questionnaire to determine demographic characteristics and previous experience in mastoidectomy. Subjects were randomized to one of two groups using a random number generator. Participant enrollment and implementation of the random allocation sequence was done by one of the authors (AC) who was not one of the study evaluators.

Subjects were video recorded performing a baseline mastoidectomy in the temporal bone laboratory setting. Subjects were randomly assigned to dissect either the left or right side as the baseline dissection by random number generator. Care was taken to capture the subjects’ hands, only; no identifying features were recorded to ensure anonymity for the subjects and blinding for the study evaluators. Subjects were given both verbal and written instructions specifically to “dissect the following temporal bone structures: tegmen, external auditory canal, sigmoid sinus, sinodural angle, antrum, short process of the incus, facial recess, chorda tympani and facial nerve.” Subjects were given a 25-min time limit in which to perform the procedure.

Upon completion of the baseline dissection, all subjects in both groups were given a textbook excerpt [21] and instructed to study the material before completing a second temporal bone dissection in the laboratory 48 h later as is standard practice for preparation for many temporal bone dissection courses. In addition, subjects in the textbook study group were given an opportunity to review the textbook material for approximately ten minutes before completing the second temporal bone dissection. Subjects in the treatment group (*n* = 6) were enrolled in the mental practice protocol. The protocol was administered to the subjects by one of the authors (AC) immediately after the first dissection. Upon completion of a brief relaxation period and introduction to the concept of mental practice, the subjects were read the mental practice script. To accommodate various learning types (i.e. visual and auditory), subjects were provided with both a written copy of the script and a set of ten detailed illustrations to read and/or view while the script was read to the subjects out loud.. These illustrations were identical to the diagrams in the textbook excerpt. Subjects were encouraged to pay attention to the visual, cognitive and kinesthetic cues emphasized in the script, and to actively imagine performing a mastoidectomy. Subjects in the treatment group were given a copy of the mental practice script to take home with them to review prior to the second mastoidectomy 48 h later.

Each subject was then video-recorded completing a second, post-intervention mastoidectomy in the temporal bone dissection laboratory. The dissection was performed at the same drilling station on the contra-lateral side of the same cadaveric specimen, as there is a high degree of correlation in temporal bone volume and surface anatomy between left and right sides of the same specimen [22]. Subjects were provided with a new set of the same sized burs for each dissection. Upon completion of the final dissection, subjects in the mental practice group also assessed the quality of the mental practice script using the standardized Mental Imagery Questionnaire-Revised (MIQ-R).

A complete set of digital recordings of all 24 temporal bone dissections was created for each independent reviewer. The order in which the reviewers were to evaluate each set of dissections was determined using a random number generator to ensure that the evaluator could not determine whether a given dissection was performed pre- or post-intervention, nor whether the subject was in the control or treatment group. Each evaluator reviewed all the dissections but in a different order. There were no deviations from the intended protocol; all subjects received the intended treatment, all subjects were analyzed for all outcome measures and there were no losses or exclusions after randomization.

Two experienced Otologists (BDW, MB) working from separate sites reviewed and coded each dissection independently, on two separate occasions separated by at least 4 weeks. The evaluators reviewed the entirety of each 25-min dissection, and were permitted to rewind or repeat their viewing of any segment of the recording at their discretion. Each video recording was evaluated using two validated, reliable instruments for evaluating mastoidectomy performed in the temporal bone lab [20]: the Task Specific Evaluation of Mastoidectomy (primary outcome) and the Global Evaluation of Mastoidectomy (secondary outcome) (Additional file 1). The Task Specific Evaluation checklist includes evaluation of seven tasks involved in completing a mastoidectomy; subjects were not required to complete the dissection of the digastric ridge, and therefore, this task was scored “not applicable” for each subject. The Global Evaluation checklist includes general evaluation of proficiency with use of equipment, flow of the surgery and a score for overall surgical performance. Before reviewing any of the digital recordings of the subjects’ mastoidectomy, the evaluators were given the opportunity to openly discuss these evaluation checklists with each other and clarify any potential sources of discrepancy. Each evaluator then worked independently to score the recorded dissections.

#### Statistical methods

Pre-intervention and post-intervention Task Specific Evaluation of Mastoidectomy, and Global Evaluation of Mastoidectomy scores were compared between the groups using a two-factor ANOVA with repeated measures test. The two evaluators were compared to each other for each of the two scores using a Spearman Rank correlation test and for correlation between their own Task Specific Evaluation of Mastoidectomy scores and Global Evaluation of Mastoidectomy scores using the Spearman Rank correlation test. For all tests a *p* value less than 0.05 was required to reject the null hypothesis.

## Results

### Development of the mental practice protocol

Table 1 provides an example of excerpts from the semi-structured focused interviews held with expert surgeons in development of the mastoidectomy mental practice script, including examples of visual and kinesthetic cues. The mental practice script was assessed prior to implementation by six OHNS Residents, PGY3 to 5. The mean overall score was 5.8 (5 = somewhat helpful; 6 = helpful) (Range: 4–7). The script was also evaluated post-implementation by the six mental practice subjects; the mean overall score was 5.3.

**Table 1:** Example excerpts from interviews and mental practice script cues

Interviewer’s prompt	Excerpt from interview transcript	Imagery cues in final Mental Practice script
“Discuss proper placement of the initial bone cuts along the posterior canal.”	“Find the spine of Henle, and basically hug the posterior ear canal…place the suction in the ear canal so you can use a kinesthetic feel of the suction and drill to see how closely they are to each other while drilling.”	*Kinesthetic:* “By placing your suction in the ear canal, you get a kinesthetic feel, tactile feedback telling you the thickness of the bone.”
“Discuss identification of the facial recess.”	“From the line through the body of the incus and inferiorly into the posterior canal, that line delineates the facial recess, what Residents are taught is to be faithful to that line.”	*Visual:* “Now, you clearly visualize a line through the body of the incus pointing inferiorly along the posterior ear canal. You start drilling, remaining faithful to this line.”

### Impact of mental practice on mastoidectomy skills

Baseline demographic characteristics and mastoidectomy experience of the subjects in each group were comparable (Table 2). Although the Mental Practice Group may have performed more mastoidectomy procedures as the first surgeon, this was countered in the control group by a greater number of procedures performed as second surgeon and a greater number of lab dissections performed. The comparability of groups is further reflected in the identical baseline pre-intervention Task Specific Evaluation of Mastoidectomy scores (Table 3).

**Table 2:** Baseline demographic characteristics and mastoidectomy experience, comparisons between groups

Variable	Group 1 (Textbook)	Group 2 (Mental Practice)
No. of subjects	6	6
Mean age (yrs)	35	31
Mean no. first surgeon procedures	17	22
Mean no. second surgeon procedures	21	13
Mean no. lab dissections	11	7
Mean no. of courses	2	2

**Table 3:** Comparison between mean total *Task Specific Evaluation of Mastoidectomy* score, Textbook Study group versus Mental Practice group

	Textbook Study Group Mean (standard deviation)	Mental Practice Group Mean (standard deviation)
Pre-intervention	40.8 (11.1)	42.3 (5.0)
Post intervention	44.2 (15.3)	46.3 (7.6)

As would be expected, both groups of subjects demonstrated improvement on the second dissection relative to the first. On total Task-Specific Evaluation for Mastoidectomy score (primary outcome), subjects in the Mental Practice group had higher post-intervention total score than the Textbook Study group but did not reach statistical significance (*p* = 0.736). Each group had a non-statistically significant higher total score post-intervention than pre-intervention (*p* = 0.182) (Table 3). Using the two-factor ANOVA with repeated measures test, there was no interaction (no significant difference between the two groups in the change in scores post-intervention) between the group and the test run (*p* = 0.922). Both groups had a slightly non-statistically significant higher score on the Total Global Evaluation of Mastoidectomy score (secondary outcome), following the intervention (*p* = 0.395). There was no statistical difference between the two groups on the Total Global Evaluation score (*p* = 0.657) (Table 4). Using the two-factor ANOVA with repeated measures test, there was no interaction between the group and the test run (*p* = 1.00); there was no significant difference between the two groups in the change in scores post-intervention.

**Table 4:** Comparison between mean total *Global Evaluation of Mastoidectomy* score, Textbook Study group versus Mental Practice group

	Textbook Study Group Mean (standard deviation)	Mental Practice Group Mean (standard deviation)
Pre-intervention	11.6 (3.3)	13.1 (1.8)
Post intervention	12.3 (5.0)	13.6 (3.0)

**Fig. 1:**
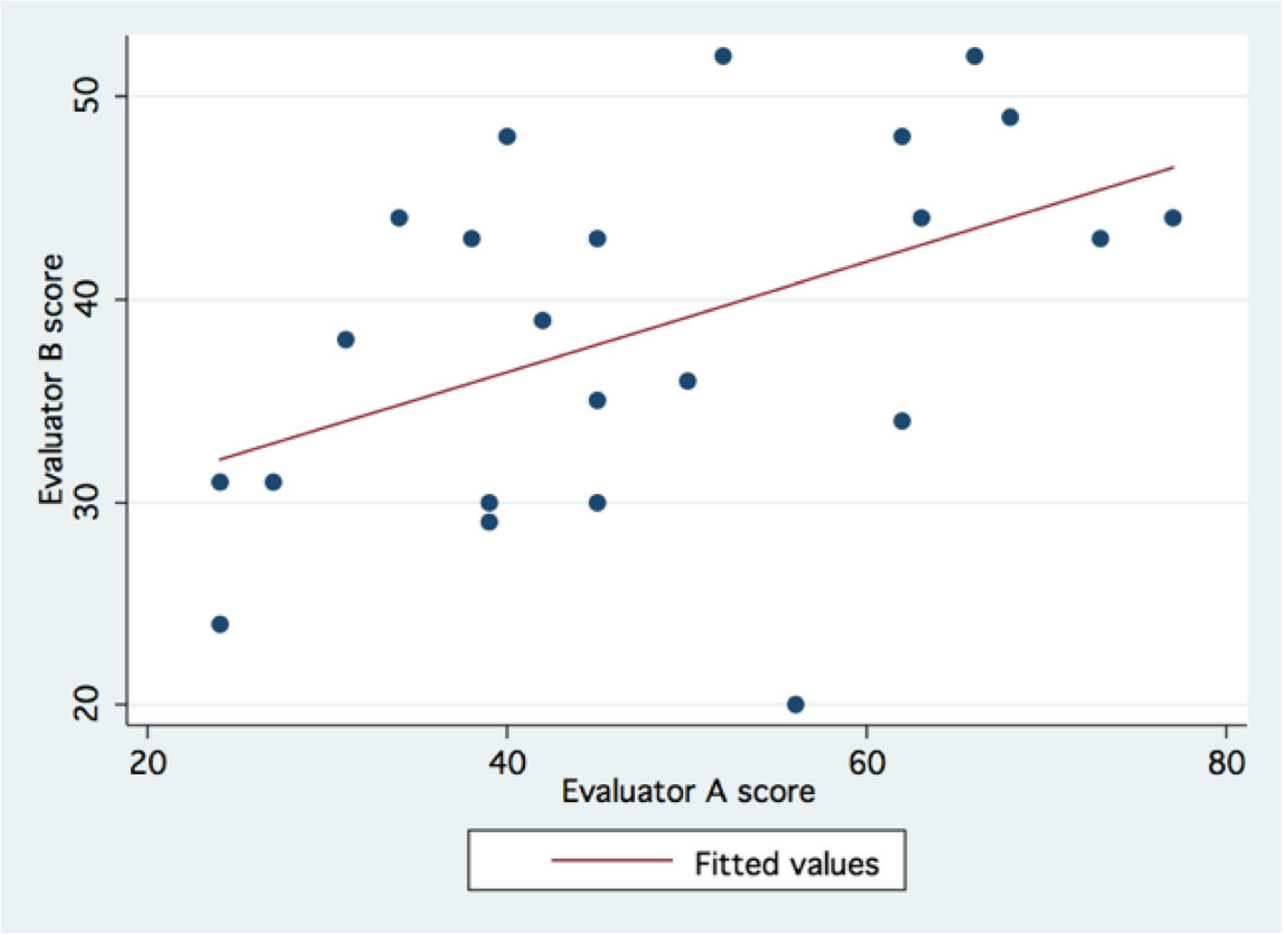
Correlation between evaluators based on total *Task Specific Evaluation of Mastoidectomy* scores

**Fig. 2:**
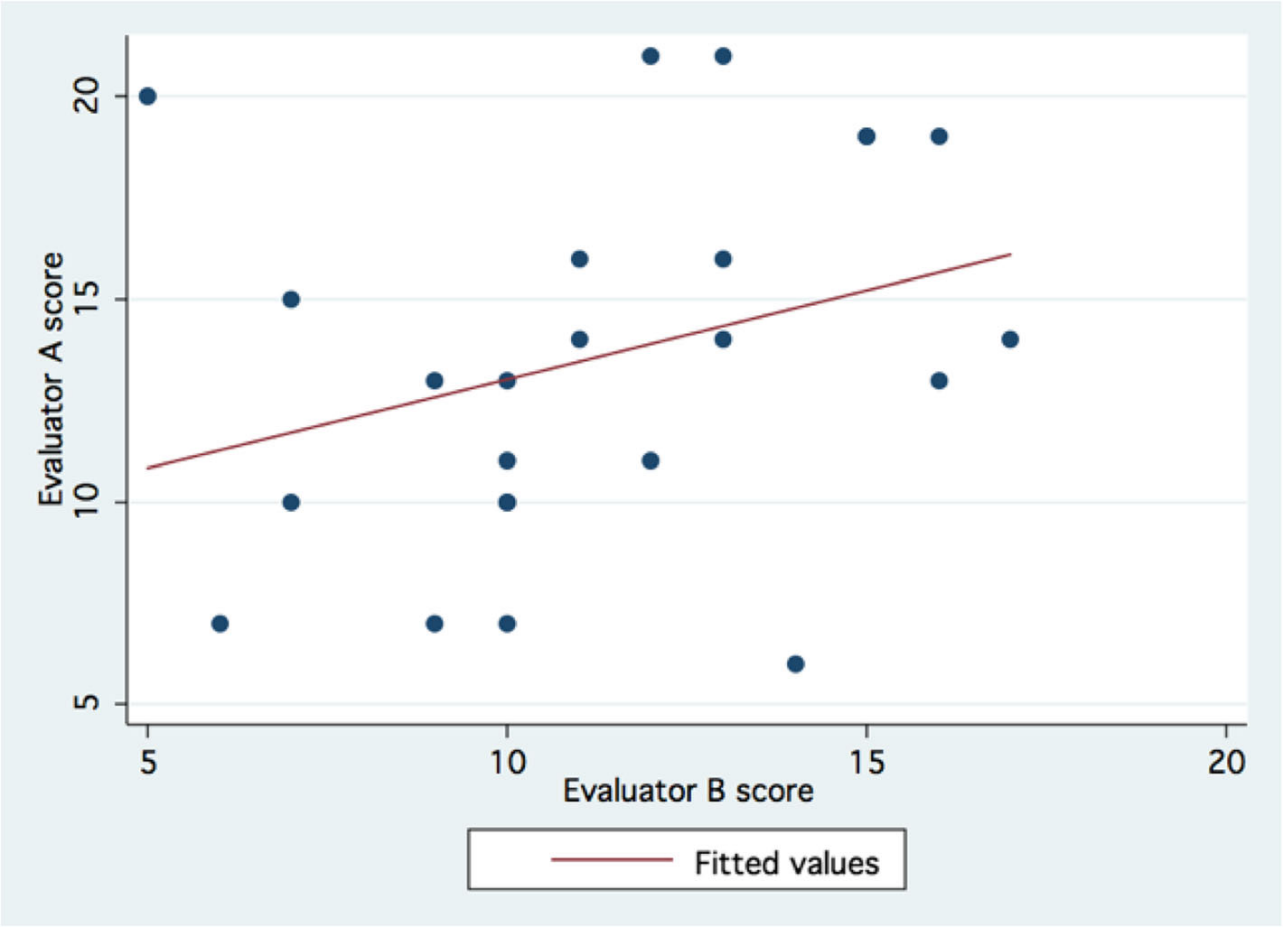
Correlation between evaluators based on total *Total Global Evaluation of Mastoidectomy* scores

**Fig. 3:**
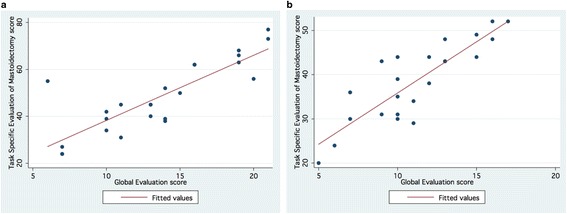
**a** Correlation between total Task Specific Evaluation of Mastoidectomy score and total Global Evaluation score for evaluator A. **b** Correlation between total Task Specific Evaluation of Mastoidectomy score and total Global Evaluation score for evaluator B

Overall, there was significant correlation (Spearman’s rho = 0.495; *p* = 0.0153) between the two evaluators on the primary outcome measure of the study, the total Task Specific Evaluation of Mastoidectomy score (Fig. 1). However, the correlation between the evaluators on the secondary outcome measure, total Global Evaluation of Mastoidectomy score was non-significant (Spearman’s rho = 0.350; *p* = 0.0852) (Fig. 2). Each evaluator showed very high consistency with statistically significant intra-evaluator correlations (Spearman’s rho = 0.809, *p* < 0.001 for Evaluator A; Spearman’s rho = 0.811, *p* < 0.001 for Evaluator B) (Fig. 3).

## Discussion

With input from three expert surgeons we developed a clear, unified and reproducible mental practice script containing visual, cognitive and kinesthetic cues, which OHNS Residents in their senior years found helpful in visualizing the surgical steps required for mastoidectomy. Mental practice may have a concurrent role by Residents along with simulation and cadaveric dissection in attainment of competency in mastoid surgery.

Recently, greater attention has been paid to the unique challenges in training Residents in mastoidectomy surgery [23]. Previously, residency training programs relied upon volume of exposure, but more recently, education theory has been applied to surgical training in order to evaluate means of skill acquisition in a more structured way. Fitts and Posner’s Theory of Motor Skill Acquisition describes three distinct phases through which a learner must pass in obtaining a new motor skill. Stage 1, the cognitive stages, is the process of intellectualizing the task, whereby performance is erratic and the procedure is carried out in distinct steps. Traditional textbook study has been an effective method to aid a learner in Stage 1, as a significant volume of knowledge must be obtained by a Resident at the outset of learning to perform for instance a mastoidectomy. Stage 2, the integrative stage, is marked by the transfer of knowledge to behavior; in Stage 3, the autonomous stage, the performance is smoother, there is no need to think about movements, and the student can concentrate on higher learning [23].

Our concern with traditional teaching methods, such as textbook learning, is that they have primarily focussed on the cognitive stage. Mental practice, however, is a teaching method developed with the fundamental goal of helping the learner perform a mastoidectomy in an integrative and autonomous manner. Put another way, we feel that mental practice fundamentally differs from traditional textbook learning methods, in that the emphasis is on mastery and autonomy of a surgical skill, rather than simple acquisition of knowledge.

There are many challenges inherent to studying the benefit of any adjunctive intervention in surgical skills acquisition. We chose two validated tools designed to evaluate trainee competency in mastoidectomy. Our selected outcome measures (Task Specific Evaluation of Mastoidectomy: Global Evaluation of Mastoidectomy), demonstrated high and statistically significant inter-rater reliability, a finding not replicated in other mental practice studies [16, 24, 25], implying generalizability of the results of this study. We failed to demonstrate a statistically significant benefit for mental practice, possibly because the study was underpowered to detect such a difference if one existed. Our sample size (*n* = 12) is similar to those of other published research studies involving Residents’ learning of mastoidectomy skills. Francis et al [26] enrolled nine Residents in their study defining milestones in mastoidectomy competency; Francis and colleagues studied 15 Residents to develop an objective assessment tool for Residents performing mastoidectomy in the operating room [27]. A larger sampling of Residents in their senior year of training would require a multi-national and probably multi-year study with additional inherent limitations. Mental practice may have greater benefit when repeated and with active engagement by the learner in a relaxed setting [26], such that if it were performed repeatedly throughout a 5-year residency training program, sequential additional improvement in performance may be seen.

Additionally, the effectiveness of mental practice in surgical skill acquisition has been corroborated in non-otologic procedures, specifically on junior Residents’ performance of a vaginal hysterectomy [24]. Few randomized controlled trials have been published regarding training adjuncts for Residents learning mastoidectomy. Greater improvement on temporal bone dissection after supervised training using a virtual reality simulator than with traditional teaching methods was demonstrated in a randomized controlled trial involving 20 trainees [11]. However, the virtual reality simulator requires significant constraints on time and financial resources, whereas mental practice is an intervention that is portable, accessible and inexpensive. Mental practice has in fact been described as, “the simulation centre in the mind [16].”

Prior mental practice research in surgery has been criticized for having a rather poorly specified imagery intervention [15]. Interventions with greater degrees of visualization exercises have demonstrated superior results. For instance, when junior Residents rehearsed performance of cystoscopy and were required to not only visualize but also describe to a surgeon-educator the steps involved in performance of cystoscopy, statistically-significantly higher scores among subjects in the mental practice group were recorded upon completion of their first cystoscopy [25]. Because the benefit of mental practice disappeared after subjects performed only one cystoscopy, the authors hypothesized that physical practice may have played a large role in cystoscopy skill acquisition, and that mental practice may have greater benefit in more complicated procedures with a larger cognitive component [25]. Arguably, performance of mastoidectomy has a larger cognitive component.

Other surgical education studies on mental practice for other surgical specialties have been criticised for not including a method to validate the mental practice script [15]. Content of this mental practice script was based on expert reviewers and analysed using standard qualitative research techniques to ensure consensus across the three experts. We then used quantitative methods to evaluate the imagery within the script, insofar as whether a Resident could see the dissection in the mind’s eye. If the description was rich enough, we believed that any surgeon, resident or consultant, ought to be able to imagine the dissection. Both the group of Residents who previewed the script and the group of Residents who applied the script in the laboratory gave it a rating between “somewhat helpful” and “helpful.” Determining means to improve these scores (for example, by improving the mental practice script itself or providing Residents more opportunities to learn and apply mental practice techniques in residency) may lead to greater benefit for the learner. A variety of mental practice protocols have been used in surgical education studies. One such protocol involved a clinical psychologist guiding subjects through a 30-min period of relaxation exercises and mental imagery immediately before completing the surgical task [16], a technique that would be difficult to incorporate into the daily routine of a surgical trainee. Our mental practice script was developed on the premise it should be easily applicable, and easily incorporated into the surgeon’s daily routine. If mental practice were to be incorporated into mainstream surgical education, it would be ideal to determine to whom, when and how often this form of learning should be ideally applied.

We employed a systematic process in development of the mental practice script, incorporating the results of interviews of three different surgeon-educators from three different training programs across Canada, hoping to be representative of the actual surgical trainee’s experience, with pearls of wisdom gleaned from multiple surgeon-educators. Educators can expect that some of the variability of effectiveness of mental practice is learner specific; just as there are different learning styles regarding auditory, visual and tactile input, there may be learners who are more adept at learning through mental practice paradigms. Our mental practice script tried to incorporate the visual, cognitive and kinesthetic cues involved in the performance of the procedure.

## Conclusions

The realities of the contemporary surgical training environment are such that effective and inexpensive learning opportunities are critical; both educators and trainees need to do more with less. Mental practice is a means of surgical training that is portable, accessible, inexpensive and safe. We offer support for further investigation and refinement of this technique for training Residents in mastoidectomy surgery. Although unlikely to replace other adjunctive modalities for surgical education, it may serve a concurrent role in surgical skills acquisition by surgeons in training.

## Additional file

Additional file 1: Checklist 1: Task-specific evaluation of mastoidectomy [22]. Checklist 2: Global evaluation of mastoidectomy [22]. (ZIP 10 kb)
