# Supplementary material for: Mental practice in postgraduate training: a randomized controlled trial in mastoidectomy skills
Source: J Otolaryngol Head Neck Surg. 2016 Sep 15;45:46. doi: 10.1186/s40463-016-0162-2 (PMC5025613; doi:10.1186/s40463-016-0162-2)
Supplement: Additional file 1: — Checklist 1: Task-specific evaluation of mastoidectomy [22]. Checklist 2: Global evaluation of mastoidectomy [22]. (ZIP 10 kb) [file 40463_2016_162_MOESM1_ESM.zip › CanJoO_Mental Practice_Checklist 1_10Mar2016.doc]

**Appendix A**

**Checklist 1: Task-specific evaluation of mastoidectomy^22^**

**Mastoidectomy Checklist Instrument**

| **Tasks** | **Unable to Perform** |  | **Performs with Minimal Prompting** |  | **Performs Easily with Good Flow** | **Not Applicable** | |
| --- | --- | --- | --- | --- | --- | --- | --- |
|  | | | | | | |  |
| 1. Initial bone cuts | | | | | | |  |
| a. Placement of superior cut | 1 | 2 | 3 | 4 | 5 | NA | |
| b. Placement of canal cut | 1 | 2 | 3 | 4 | 5 | NA | |
| 2. Defining anatomic limits | | | | | | |  |
| a. Identification and definition of tegmen | 1 | 2 | 3 | 4 | 5 | NA | |
| b. Sharpen posterior EAC cortex | 1 | 2 | 3 | 4 | 5 | NA | |
| c. Define sigmoid sinus and sino-dural angle | 1 | 2 | 3 | 4 | 5 | NA | |
| 3. Open antrum | | | | | | |  |
| a. Deepen dissection at sino-dural angle | 1 | 2 | 3 | 4 | 5 | NA | |
| b. Open antrum from posterior to anterior | 1 | 2 | 3 | 4 | 5 | NA | |
| c. Reveal short process of incus (atraumatic) | 1 | 2 | 3 | 4 | 5 | NA | |
| 4. Digastric dissection | | | | | | |  |
| a. Define cephalic edge digastric muscle | 1 | 2 | 3 | 4 | 5 | NA | |
| b. Follow to SMF | 1 | 2 | 3 | 4 | 5 | NA | |
| 5. Thin posterior EAC cortex (translucent) | | | | | | |  |
| a. View posterior EAC *en face* | 1 | 2 | 3 | 4 | 5 | NA | |
| b. Use side/front of appropriate bur | 1 | 2 | 3 | 4 | 5 | NA | |
| c. Saucerization | 1 | 2 | 3 | 4 | 5 | NA | |
| 6. Open facial recess | | | | | | |  |
| a. Even removal of infra-labyrinthine bone | 1 | 2 | 3 | 4 | 5 | NA | |
| b. Medial thinning of EAC cortex | 1 | 2 | 3 | 4 | 5 | NA | |
| c. Identify and preserve VII and chorda | 1 | 2 | 3 | 4 | 5 | NA | |
| d. Identify ME anatomy | 1 | 2 | 3 | 4 | 5 | NA | |
| e. Decompress facial nerve to SMF | 1 | 2 | 3 | 4 | 5 | NA | |
| 7. Posterior atticotomy | | | | | | |  |
| a. Thin superior EAC cortex | 1 | 2 | 3 | 4 | 5 | NA | |
| b. Thin anterior tegmen | 1 | 2 | 3 | 4 | 5 | NA | |
| c. Remove intervening bone | 1 | 2 | 3 | 4 | 5 | NA | |
| d. Identify epitympanic anatomy to supratubal recess  TOTAL SCORE: | 1 | 2 | 3 | 4 | 5 | NA | |
