# Supplementary material for: Mental practice in postgraduate training: a randomized controlled trial in mastoidectomy skills
Source: J Otolaryngol Head Neck Surg. 2016 Sep 15;45:46. doi: 10.1186/s40463-016-0162-2 (PMC5025613; doi:10.1186/s40463-016-0162-2)
Supplement: Additional file 1: — Checklist 1: Task-specific evaluation of mastoidectomy [22]. Checklist 2: Global evaluation of mastoidectomy [22]. (ZIP 10 kb) [file 40463_2016_162_MOESM1_ESM.zip › CanJoO_Mental Practice_Checklist 2_10Mar2016.doc]

**Checklist 2: Global evaluation of mastoidectomy^22^**

**Table II. The Global Instrument**

| Use of otologic drill | 1 | 2 | 3 | 4 | 5 |
| --- | --- | --- | --- | --- | --- |
|  | Chooses inappropriate bur and/or repeatedly awkward use of drill |  | Chooses appropriate bur and occasionally awkward use of drill |  | Uses appropriate bur and drill effortlessly |
| Use of microscope | 1 | 2 | 3 | 4 | 5 |
|  | Repeatedly inappropriate position, magnification, or focus |  | Competent use of microscope but occasional inappropriate position and magnification |  | Optimal visualization with appropriate microscope use |
| Respect for surgical limits | 1 | 2 | 3 | 4 | 5 |
|  | Uses unnecessary force or caused damage by inappropriate use of instruments |  | Careful handling of tissue but occasional inadvertent damage to tissue |  | Consistently handled tissues appropriately with minimal damage |
| Time and motion | 1 | 2 | 3 | 4 | 5 |
|  | Many unnecessary moves |  | Efficient time/motion and maximum efficiency |  | Clear economy of movement |
| Flow of operation | 1 | 2 | 3 | 4 | 5 |
|  | Frequently stopped and unsure of next move |  | Some forward planning with reasonable progression |  | Obviously planned course of operation with effortless flow |
| Overall surgical performance | 1 | 2 | 3 | 4 | 5 |
| TOTAL: | Poor |  | Performs majority of surgery acceptably |  | Outstanding |
